# Early vascular responses to everolimus-eluting cobalt–chromium stent in the culprit lesions of st-elevation myocardial infarction: results from a multicenter prospective optical coherence tomography study (MECHANISM-AMI 2-week follow-up study)

**DOI:** 10.1007/s12928-017-0507-4

**Published:** 2018-01-09

**Authors:** Yoshihiro Morino, Daisuke Terashita, Hiromasa Otake, Tatsuo Kikuchi, Tetsuya Fusazaki, Nehiro Kuriyama, Takahide Suzuki, Yoshiaki Ito, Kiyoshi Hibi, Hiroyuki Tanaka, Shozo Ishihara, Toru Kataoka, Takashi Morita, Yoritaka Otsuka, Takatoshi Hayashi, Kengo Tanabe, Toshiro Shinke

**Affiliations:** 1 Division of Cardiology, Department of Internal Medicine, Iwate Medical University, 19-1 Uchimaru, Morioka, Iwate 020-8505 Japan; 2 Kobe University Graduate School of Medicine, Kobe, Hyogo Japan; 3 Edogawa Hospital, Edogawa, Tokyo, Japan; 4 Miyazaki Medical Association Hospital, Miyazaki, Miyazaki Japan; 5 Hokkaido Welfare Federation of Agricultural Cooperative Engaru Kosei General Hospital, Monbetsugun, Hokkaido, Japan; 6 Saiseikai Yokohama-City Eastern Hospital, Yokohama, Kanagawa Japan; 7 Yokohama City University Medical Center, Yokohama, Kanagawa Japan; 8 Tokyo Metropolitan Tama Medical Center, Fuchu, Tokyo Japan; 9 Mimihara General Hospital, Sakai, Osaka Japan; 10 Bell Land General Hospital, Sakai, Osaka Japan; 11 Osaka General Medical Center, Osaka, Osaka Japan; 12 Fukuoka Wajiro Hospital, Fukuoka, Fukuoka Japan; 13 Hyogo Prefectural Awaji Medical Center, Sumoto, Hyogo Japan; 14 Mitsui Memorial Hospital, Tokyo, Japan

**Keywords:** Drug-eluting stent, Myocardial infarction, Optical coherence tomography, Thrombus

## Abstract

The use of cobalt–chromium everolimus-eluting stents (CoCr-EES) for ST-segment elevation myocardial infarction (STEMI) reduces the incidence of stent thrombosis compared with bare metal stents, and a substantial difference is apparent in the initial 2 weeks. However, vascular behavior during this early period remains unclear. This was a prospective study (MECHANISM-AMI-2W) to investigate early vascular responses in STEMI patients immediately after CoCr-EES implantation and at 2-week follow-up using frequency domain-optical coherence tomography (FD-OCT). The study enrolled 52 patients (age 63.7 ± 11.7 years, male 85.0%), of whom 44 patients were available for complete serial FD-OCT analyses. Both % uncovered struts and % malapposed struts were improved at 2-week follow-up (63 ± 20 vs. 21 ± 14%, *p* < 0.0001 and 7.3 ± 9.0 vs. 4.7 ± 5.9%, *p* = 0.005, respectively). Thrombus was decreased, with significant changes in longitudinal length to stent (28.8 ± 27.7 vs. 18.1 ± 20.2%, *p* = 0.0001) and maximal area (0.93 ± 0.84 vs. 0.65 ± 0.63 mm^2^, *p* = 0.034). As a result, the average lumen area was significantly larger at 2 weeks (6.49 ± 1.82 vs. 6.71 ± 1.89 mm^2^, *p* = 0.048, respectively). The number of dissection flaps was lower (0.86 ± 1.11 vs. 0.52 ± 0.90%, *p* = 0.024). In conclusion, this study showed early vascular responses to CoCr-EES for STEMI lesions—including a significant reduction of thrombus—that resulted in lumen enlargement, earlier progression of strut coverage, and improvements in strut apposition and dissection. The combination of these factors may therefore be responsible for the safety of CoCr-EES within the initial 2 weeks.

## Introduction

Percutaneous coronary intervention (PCI) is an established method to improve the prognosis of patients with ST-segment elevation myocardial infarction (STEMI). A meta-analysis of 15 randomized controlled trials for the treatment of STEMI demonstrated that the use of first-generation drug-eluting stents decreased the need for repeated revascularization, but slightly increased the risk of very late stent thrombosis compared with bare metal stents [1]. Furthermore, a pathological study indicated that delayed vessel healing at the culprit site of stent implantation may increase the risk of stent thrombosis [2]. Thus, the overall benefits of drug-eluting stents for STEMI were controversial for almost a decade.

However, since the introduction of second-generation drug-eluting stents with improved polymers and thinner struts, which are expected to enhance strut endothelialization, inhibit thrombus formation [3], and attenuate local inflammation, there has been a gradual change in the therapeutic landscape. Recent trials have demonstrated that stent thrombosis was less frequent with a cobalt–chromium everolimus-eluting stent (CoCr-EES) than a bare metal stent [4, 5], with substantial differences observed within the initial 2 weeks after implantation [4]. However, the safety of CoCr-EES in STEMI patients remains unclear due to the lack of information on the underlying mechanisms of local vascular healing during the early phase. Insertion of a foreign metal body into thrombus-rich segments may be expected to increase the risk of local thrombogenicity. To investigate the vascular processes after stent implantation, frequency domain-optical coherence tomography (FD-OCT) is an ideal tool because it enables high-resolution imaging of in-stent structures, including quantification of strut coverage [6], the degree of strut malapposition [7], and volume of thrombus [8–10], which are considered to be continuous ST risks. Therefore, this study was designed to elucidate the early phase vascular responses after CoCr-EES, focusing on serial changes of clinically relevant intravascular findings for culprit lesions of STEMI using serial FD-OCT analyses.

## Methods

### Study design and population

The MECHANISM-AMI-2W (Multicenter Comparison of Early and Late Vascular Responses to Everolimus-eluting cobalt–CHromium Stent and platelet AggregatioN studIeS for TreatMent of Acute Myocardial Infarction 2 weeks FD-OCT follow-up) study was planned for specific purposes (ClinicalTrials.gov ID: NCT02014753, UMINID: UMIN000012616). Prior to MECHANISM-AMI-2W, a pilot study (MECHANISM-Pilot) was conducted to determine the optimal FD-OCT parameters [11]. The MECHANISM-AMI-2W study enrolled patients with STEMI who underwent FD-OCT immediately after primary PCI and were eligible for follow-up FD-OCT assessment at 2 weeks after implantation. Patients were recruited between April 2014 and May 2015 from 13 Japanese institutions. STEMI was defined in accordance with the Third Universal Definition of Myocardial Infarction [12]. Exclusion criteria were: cardiogenic shock, culprit lesion of left main coronary artery, reference vessel diameter < 2.0 or ≥ 4.5 mm, chronic kidney disease as indicated by serum creatinine > 2.0 mg/dl, maintenance hemodialysis, comorbid cancer with expected survival < 2 years, scheduled surgery within 3 months, history of adverse reactions to aspirin or clopidogrel, warfarin intake before STEMI onset, age < 20 years, pregnancy, and STEMI at prior stented segment.

Culprit lesions were treated with either one or two CoCr-EES (Xience Prime/Xpedition, Abbott vascular, Santa Clara, CA, USA), following thrombus aspiration therapy if needed. Concurrent use of distal protection devices was dependent on the operator’s discretion. Immediately after the procedure, FD-OCT image acquisition was performed throughout the stented segment, with a margin segment ≥ 5 mm from the culprit lesion. A loading dose of aspirin (81–200 mg) and either clopidogrel (300 mg) or prasugrel (20 mg) (the standard Japanese loading doses) was administered before PCI. Dual antiplatelet therapy with aspirin and thienopyridine was recommended for 12 months after PCI. This study complies of the Declaration of Helsinki. The study protocol was approved by the Ethical Committee of each participating institution. All patients provided written informed consent before inclusion.

### Coronary angiography and FD-OCT image acquisition

Coronary imaging was performed immediately after PCI, and after 2 weeks (14 ± 4 days) during staged PCI for residual stenotic lesions or follow-up angiography for pre-discharge evaluation. Intracoronary injection of nitroglycerin (isosorbide dinitrate or nitroglycerin) was administered. Angiograms were obtained from a standard series of multiple projections. All angiographic images were stored on a CD/DVD-ROM for offline analysis. FD-OCT images were acquired with an OCT system (ILUMIEN or ILUMIEN OPTIS) and an FD-OCT catheter (Dragonfly/Dragonfly JP/Dragonfly OPTIS) (St. Jude Medical, St. Paul, MN, USA). All FD-OCT images of the target lesion site (coronary segment from ≥ 5 mm distal and 5 mm proximal to the target lesion) were recorded with an automatic pullback system. Although the use of a contrast agent as a flushing liquid was recommended, lactated Ringer’s solution or low-molecular weight dextran L were also acceptable. FD-OCT images were digitally stored and submitted for offline analyses.

### Image analysis and definitions and study endpoints

Quantitative coronary angiography was performed at an independent core laboratory (Cardiocore Japan, Tokyo, Japan) with a standard software package (QAngio XA; Medis medical imaging systems, Leiden, The Netherlands). The stented segment and 5-mm peri-stent segments were analyzed using a standard technique. FD-OCT image analysis was performed by an independent core laboratory (Kobe Cardiovascular Core Laboratory, Kobe University Graduate School of Medicine, Kobe, Japan) using ILIMIEN proprietary software (St. Jude Medical, St. Paul, MN, USA).

Quantitative FD-OCT analyses were performed with every 1-mm interval for the assessment of lumen, stent, and intra-stent tissue area (stent area–lumen area). Intra-stent tissue thickness was measured from the lumen border to the center of the strut blooming. Struts with intra-stent tissue thickness equal to 0 μm were defined as uncovered struts. A strut that was partially covered with tissue was classified as an uncovered strut. A maximum distance of > 108 μm between the center reflection of the strut and adjacent vessel surface was defined as malapposed struts.

**Fig. 1:**
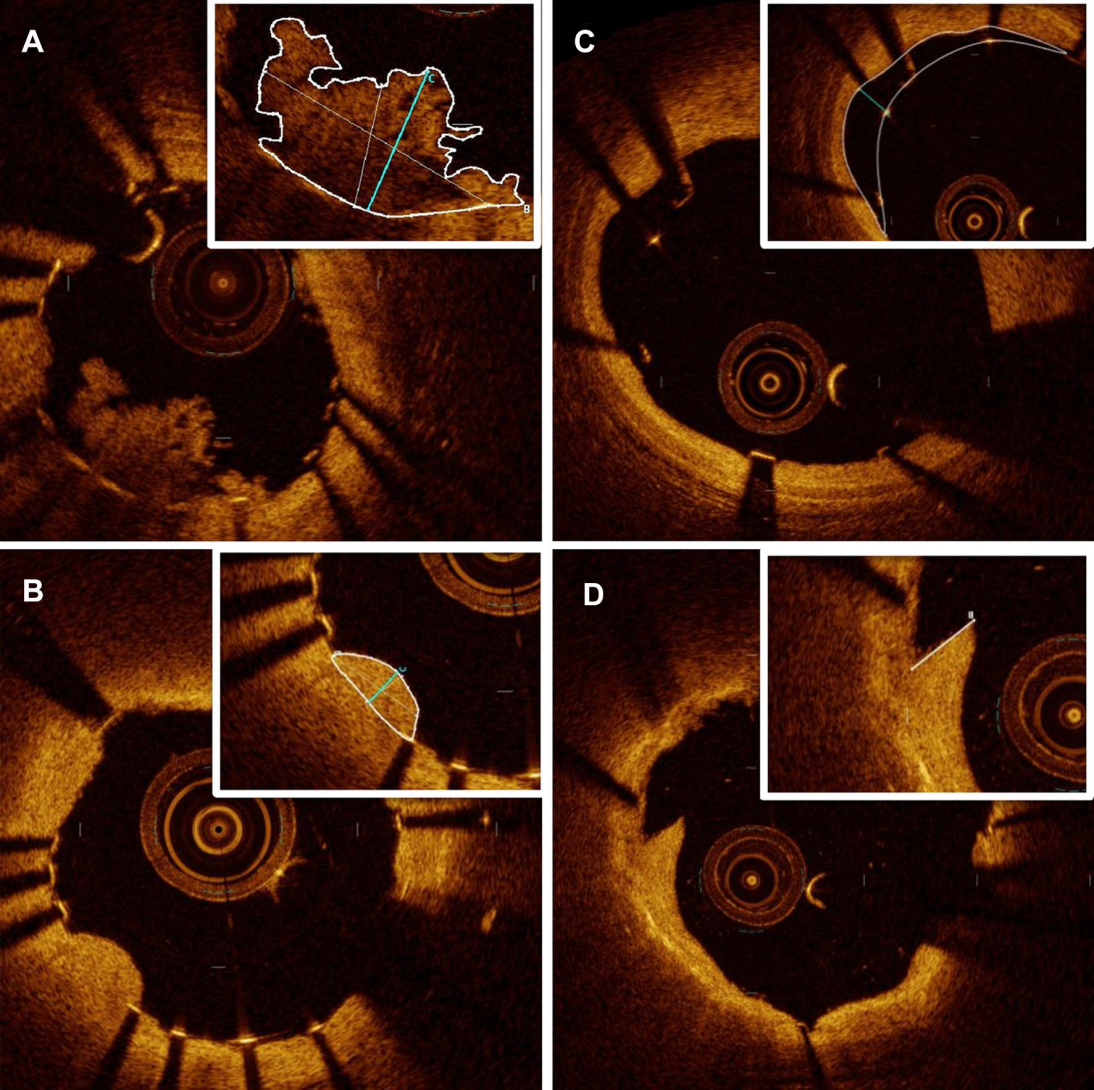
Representative quantitative parameters of in-stent findings, including thrombus (**a**), prolapse (**b**), malapposition (**c**), and dissection (**d**). Corresponding parameters (area, height/depth/flap length, longitudinal length) were measured for each thrombus or segment

Qualitative FD-OCT analyses was performed for every single frame to assess (1) thrombus, a mass ≥ 250 μm in height with an irregular surface, protrusion into the lumen, and significant attenuation behind the mass; (2) prolapse, a signal-rich protruding tissue ≥ 250 μm in height, located between the struts with a smooth surface and no attenuation; (3) intra-stent tissue, combinations of entire intra-stent tissue between lumen and stent, and (4) dissection, evidence of flaps ≥ 200 μm in height within the stent segment and stent margin, according to the published methods [13, 14]. Maximal area, maximal height or depth, and longitudinal length were measured for each mass or segment individually (Fig. 1). Total length was defined as the sum of the longitudinal length of each composition. Each % longitudinal length to stent was calculated as: the number of cross-sections with thrombus/prolapse/malapposition/dissection × 100/the total number of cross-sections within the stented segment.

The primary endpoint was % uncovered strut. Secondary endpoints included the representative described FD-OCT parameters of thrombus, prolapse, strut coverage/malapposition, and dissection, also corresponding to them.

### Statistical analyses

All statistical analysis was performed with SPSS software, version 23.0 (SPSS, Inc., Chicago, IL, USA). Normally distributed continuous variables are expressed as means ± standard deviation (SD). Non-normally distributed continuous variables are expressed as medians and ranges. Categorical variables are expressed as numbers and percentages. Fisher’s exact test or paired t-tests were performed for comparisons between post-procedure and 2 weeks follow-up. A *p* value < 0.05 was considered to denote a statistically significant difference.

## Results

### Baseline characteristics and clinical outcomes within initial 2 weeks

The baseline, lesion and procedural characteristics of the study population are shown in Table 1. A total of 52 patients (age 63.7 ± 11.7 years, male 85.0%) were enrolled with high frequencies of diabetes mellitus (42.3%) and smoking (50.0%). Following thrombus aspiration in 80.8% of patients, 1.15 ± 0.36 stents were used. Two patients (3.8%) received chronic antiplatelet therapy with aspirin prior to the onset of STEMI. All patients started to receive thienopyridine from this event and continued dual antiplatelet therapy, primarily with clopidogrel (98.1%), for 2 weeks. Neither major adverse events nor target vessel revascularizations occurred within 2 weeks.

**Table 1:** Baseline clinical, lesion, procedural characteristics (*n* = 52)

Age, years	63.7 ± 11.7
Male gender, *n* (%)	44 (85.0)
Height, cm	165.6 ± 9.3
Body weight, kg	64.3 ± 12.7
Diabetes, *n* (%)	22 (42.3)
Hypertension, *n* (%)	32 (61.5)
Dyslipidemia, *n* (%)	42 (80.8)
eGFR < 60, *n* (%)	11 (21.1)
Smoking, *n* (%)	26 (50.0)
Family history, *n* (%)	9 (17.3)
Culprit vessel, *n* (%)	
LAD, RCA, LCx	21 (40.4), 21 (40.4), 10 (19.2)
Angiographic reference diameter, mm	2.78 ± 0.56
Diameter stenosis, %	100 (1–100)
TIMI-flow, *n* (%)	
3, 2, 1, 0	1 (1.9), 11 (21.2), 6 (11.5), 34 (65.4)
Bifurcated lesions, *n* (%)	23 (44.2)
AHA/ACC lesion type, *n* (%)	
A, B1, B2, C	0 (0), 2 (3.8), 39 (75.0), 11 (21.2)
Door to balloon time, min	64 (29–276)
Used stent number, *n*	1.15 ± 0.36
Used stent diameter, mm	3.02 ± 0.39
Final maximum inflation pressure, atm	15.0 ± 3.4
Aspiration catheter uses, *n* (%)	42 (80.8)
Peak CPK, mg/dl	1351 (109–7526)
Peak CPK-MB, mg/dl	105 (4–445)
Hemoglobin, mg/dl	14.3 ± 1.8
Chronic antiplatelet use prior to STEMI, *n* (%)	
Aspirin, clopidogrel, prasugrel	2 (3.8), 0 (0%), 0 (0)
Dual antiplatelet therapy at 2 weeks, *n* (%)	52 (100)
Thienopyridine use, *n* (%)	
Clopidogrel, prasugrel	51 (98.1), 1 (1.9)
Anticoagulation drugs use	3 (6.5)

*eGFR* estimated glomerular filtration rate, *LAD* left anterior descending artery, *RCA* right coronary artery, *LCx* left circumflex artery

### Qualitative and quantitative coronary angiography

Table 2 demonstrates the findings of quantitative and qualitative coronary angiography. Thrombolysis in Myocardial Infarction (TIMI) grade 3 flow was achieved in 88.5% of patients. The angiographic reference diameter was 2.88 ± 0.53 mm at post-procedure. There were no statistical differences in angiographic parameters between post-procedure and 2 weeks follow-up except in-lesion reference diameter.

**Table 2:** Quantitative and quantitative coronary angiography

	Post-procedure	2-week follow-up	*p* value
TIMI grade, *n* (%)			
3, 2, 1, 0	46 (88.5), 5 (8.6), 1 (2.0), 0 (0)	41 (80.4), 10 (19.6), 0 (0), 0 (0)	0.238
In-lesion			
Reference diameter, mm	2.88 ± 0.53	2.78 ± 0.52	0.037
MLD, mm	2.23 ± 0.55	2.14 ± 0.59	0.129
% Diameter stenosis, %	22.7 ± 11.1	23.8 ± 11.8	0.552
In-stent			
Reference diameter, mm	2.93 ± 0.49	2.89 ± 0.48	0.095
MLD, mm	2.53 ± 0.46	2.49 ± 0.42	0.461
% DS, %	13.8 ± 9.8	13.5 ± 9.4	0.860
Stent length, mm	20.1 ± 6.6	20.0 ± 6.6	0.582
Proximal margin			
MLD, mm	2.75 ± 0.58	2.68 ± 0.54	0.202
% DS, %	11.8 ± 8.6	11.8 ± 8.6	0.867
Distal margin			
MLD, mm	2.26 ± 0.59	2.17 ± 0.58	0.118
% DS, %	16.1 ± 11.5	17.9 ± 13.3	0.244

*MLD* minimal lumen diameter, *% DS* % diameter stenosis

### Overall FD-OCT analyses

**Fig. 2:**
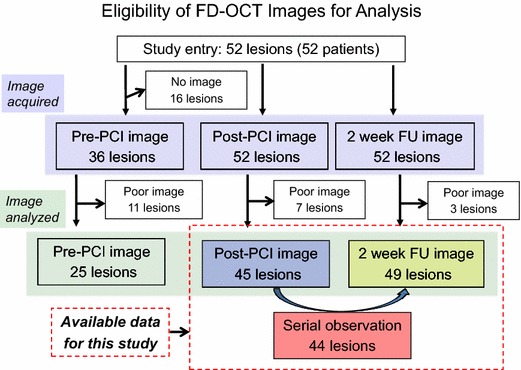
Schematic flow chart of case eligibility. Post-procedural images from 45 lesions, 2-week follow-up images from 49 lesions, and serial images from 44 cases were analyzed

Figure 2 depicts the study flow and lesions eligible for analysis. Among the 52 patients enrolled, complete FD-OCT assessment was performed in 45 lesions at post-procedure and 49 lesions at 2-week follow-up. The primary endpoint of % uncovered struts was 20.7 ± 13.7% (of 49 patients). The other general quantitative FD-OCT variables based on these entire patients (*n* = 45 at post-procedure and *n* = 49 at 2-weeks follow-up) are summarized in Table 3.

**Table 3:** Overall FD-OCT assessments at post PCI and at 2 weeks follow-up

Variables	Post-procedure (*n* = 45)	2 weeks follow-up (*n* = 49)
Visual assessments		
No. of cross-section, *n*	24.4 ± 7.4	24.7 ± 7.4
No. of struts, *n*	219 ± 76	230 ± 75
No of malapposed struts, *n*	15.9 ± 21.7	11.1 ± 15.1
% Malapposed struts, %	7.2 ± 9.0	4.7 ± 5.7
No. of uncovered struts, *n*	143 ± 68	49 ± 38
% uncovered struts, %	64.0 ± 19.7	20.7 ± 13.7
Quantitative measurements		
Average stent length, mm	23.2 ± 7.5	23.2 ± 7.4
Average stent area, mm^2^	6.65 ± 1.82	6.74 ± 1.92
Minimum stent area, mm^2^	5.36 ± 1.47	5.41 ± 1.62
Maximum stent area, mm^2^	8.07 ± 2.57	8.12 ± 2.67
Average stent diameter, mm	2.87 ± 0.40	2.89 ± 0.42
Average lumen area, mm^2^	6.50 ± 1.80	6.72 ± 1.90
Minimum lumen area, mm^2^	4.97 ± 1.54	5.21 ± 1.58
Maximum lumen area, mm^2^	8.40 ± 2.58	8.53 ± 2.67
Average lumen diameter, mm	2.83 ± 0.40	2.88 ± 0.41
Average intra-stent tissue area, mm^2^	0.33 ± 0.41	0.21 ± 0.18
Average intra-stent tissue thickness, mm	0.031 ± 0.038	0.038 ± 0.015
Minimum intra-stent tissue thickness, mm	0	0
Maximum intra-stent tissue thickness, mm	0.39 ± 0.29	0.28 ± 0.16

*PCI* percutaneous coronary intervention, intra-stent tissue thickness; distance from strut to lumen surface

### In-depth serial FD-OCT assessments

**Fig. 3:**
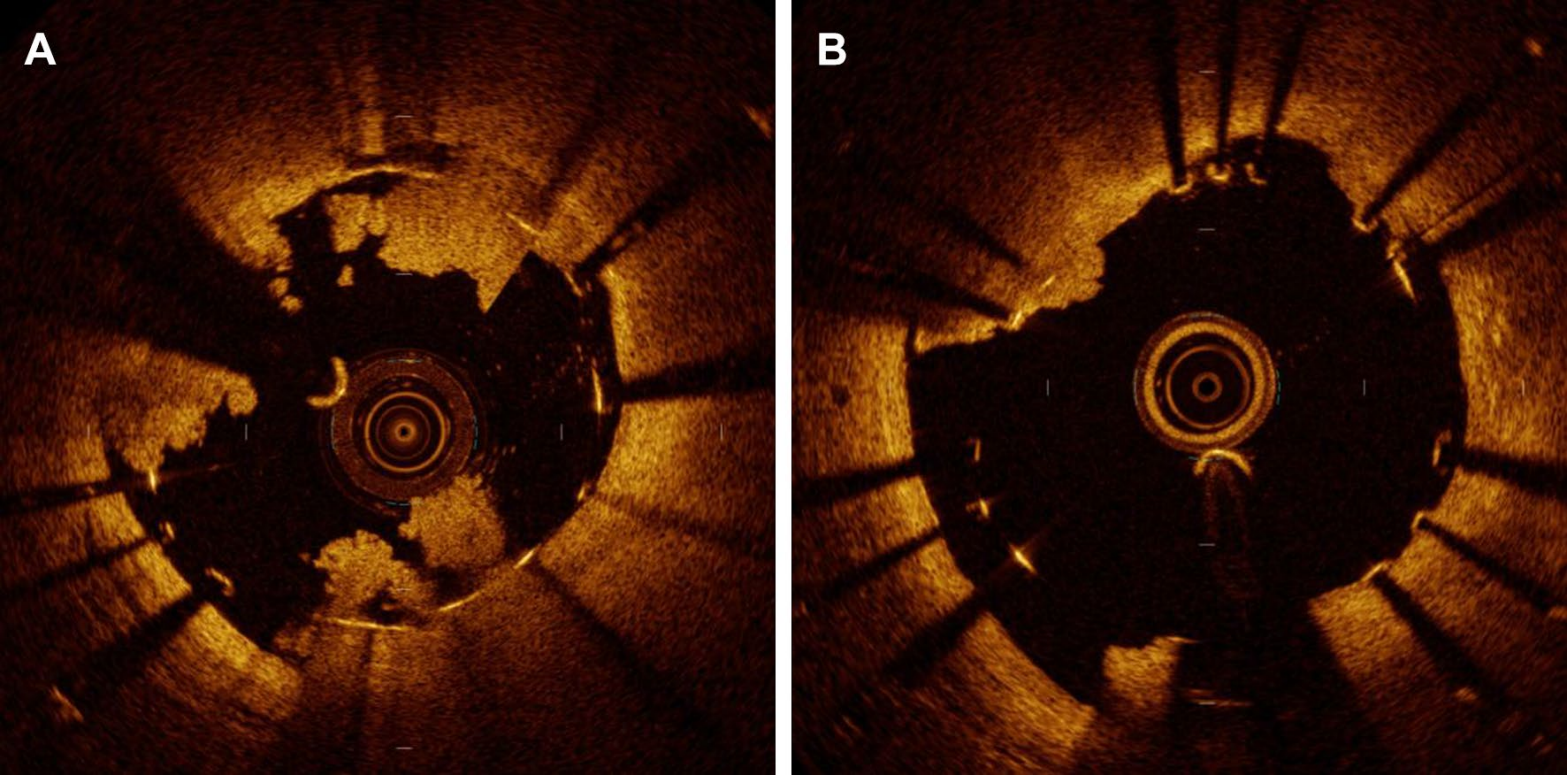
Serial FD-OCT images at post-procedure (**a**) and 2-week follow-up (**b**). In-stent thrombus was significantly decreased at 2-week follow-up in the representative cross-sections

**Fig. 4:**
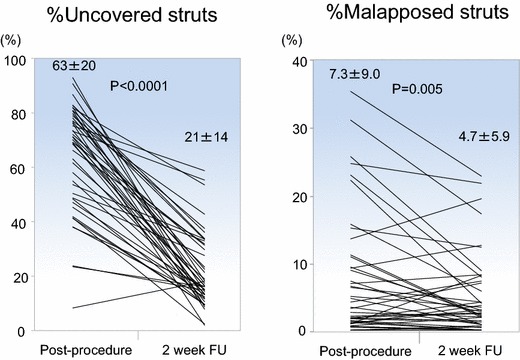
Individual plots of serial changes in % uncovered struts and % malapposed struts between post-procedure and 2-week follow-up

Table 4 displays the results of serial quantitative FD-OCT analysis. A total of 44 lesions were available for serial analyses. A representative case example of serial FD-OCT cross-sections is shown in Fig. 3. There were significant improvements in uncovered struts and malapposed struts from post-procedure to 2-week follow-up, with decreases from 63 ± 20 to 21 ± 14% (*p* < 0.0001) and 7.3 ± 9.0 to 4.7 ± 5.9% (*p* = 0.005), respectively. Their individual serial changes are plotted in Fig. 4.

**Table 4:** In-depth serial quantitative FD-OCT analysis of the stented culprit lesions (*n* = 44)

Variables	Post-procedure	2-week follow-up	*p* value
No. of cross-section, *n*	24.5 ± 7.5	24.6 ± 7.5	0.41
No. of struts, *n*	220 ± 76	227 ± 75	0.03
% Malapposed struts, %	7.3 ± 9.0	4.7 ± 5.9	0.005
% Uncovered struts, %	63 ± 20	21 ± 14	< 0.0001
Average stent length, mm	23.3 ± 7.5	23.2 ± 7.5	0.68
Average lumen area, mm^2^	6.49 ± 1.82	6.71 ± 1.89	0.048
Minimum lumen area, mm^2^	4.96 ± 1.56	5.27 ± 1.56	0.040
Average intra-stent tissue area, mm^2^	0.34 ± 0.42	0.21 ± 0.19	0.016
% intra-stent tissue area, %	4.9 ± 6.0	3.1 ± 2.5	0.024
Average intra-stent tissue thickness, mm	0.031 ± 0.038	0.038 ± 0.016	0.18
Maximum intra-stent tissue thickness, mm	0.39 ± 030	0.28 ± 0.17	0.015
Serial comparison of in-stent findings			
Thrombus			
Presence of thrombus, *n* (%)	43 (98)	37 (84)	0.029
No. of thrombus mass (≥ 250 μm), *n*	2.9 ± 1.7	1.7 ± 1.6	0.0001
Total longitudinal length, mm	6.73 ± 5.60	4.32 ± 5.02	0.0001
% Longitudinal length to stent, %	28.8 ± 22.7	18.1 ± 20.2	0.0001
Maximum area, mm^2^	0.93 ± 0.87	0.65 ± 0.63	0.034
Average maximum height of each mass, mm	0.49 ± 0.21	0.37 ± 0.27	0.0036
Prolapse			
No. of prolapse (≥ 250 μm), *n*	0.95 ± 1.0	0.52 ± 0.82	0.0116
Total longitudinal length, mm	1.36 ± 1.65	0.80 ± 1.59	0.0558
% Longitudinal length to stent, %	6.0 ± 7.1	3.9 ± 7.5	0.10
Maximum area, mm^2^	0.22 ± 0.25	0.11 ± 0.22	0.0245
Average maximum height of each prolapse, mm	0.20 ± 0.19	0.13 ± 0.22	0.1193
Malapposition			
No. of malapposed segment, *n*	2.3 ± 2.1	2.3 ± 2.0	0.85
Total longitudinal length, mm	3.94 ± 4.42	4.30 ± 5.48	0.50
% Longitudinal length to stent, %	16.5 ± 15.6	17.4 ± 18.8	0.629
Maximum area, mm^2^	0.72 ± 0.72	0.73 ± 0.73	0.870
Average maximum area of each segment, mm^2^	0.56 ± 0.60	0.52 ± 0.46	0.565
Average maximum depth of each segment, mm	0.25 ± 0.13	0.28 ± 0.16	0.277
Dissection			
No. of dissections (flap ≥ 200 μm), *n*	0.86 ± 1.11	0.52 ± 0.90	0.024
Total longitudinal length, mm	0.73 ± 1.12	0.58 ± 1.17	0.310
% Longitudinal length to stent, %	3.0 ± 7.0	2.3 ± 4.4	0.368
Transverse length of flap arm, mm	0.22 ± 0.24	0.16 ± 0.25	0.251
Proximal edge dissection^a^, *n* (%)	3.0 (6.8)	0 (0)	0.121
Distal edge dissection^b^, *n* (%)	3.0 (7.3)	3.0 (6.2)	0.63

*No.* number

^a^44 for post-procedure vs. 48 for 2-week follow-up

^b^41 for post-procedure vs. 48 for 2-week follow-up

**Fig. 5:**
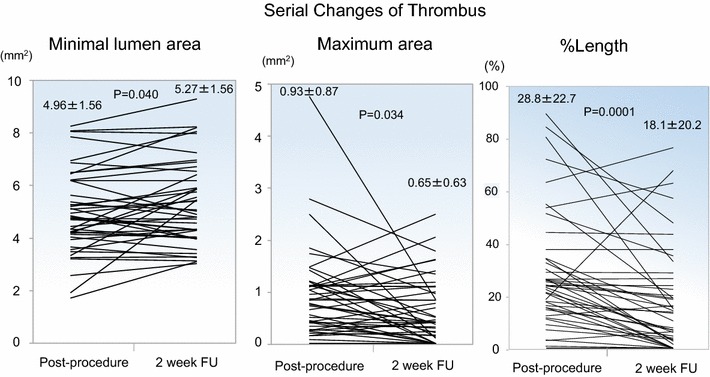
Individual plots of serial changes in minimal lumen area and thrombus (maximum area and % length) within stent between post-procedure and 2-week follow-up

The number of thrombus masses ≥ 250 μm was significantly decreased. The majority of quantitative parameters of intra-stent thrombus, including longitudinal length, height, and area, were significantly reduced (% longitudinal length to stent: 28.8 ± 22.7 vs. 18.1 ± 20.2%, *p* = 0.0001; maximum area 0.93 ± 0.87 vs. 0.65 ± 0.63 mm^2^, *p* = 0.034). A similar trend was observed for prolapse. As a consequence, the minimum and average lumen areas were larger at 2 weeks (4.96 ± 1.56 vs. 5.27 ± 1.56 mm^2^, *p* = 0.040 and 6.49 ± 1.82 vs. 6.71 ± 1.89 mm^2^, *p* = 0.048, respectively). Individual serial changes in minimal lumen area, maximum thrombus area, and percent longitudinal length of thrombus from post-procedure to 2-week follow-up are plotted in Fig. 5. The number of dissection flaps > 200 μm was also significantly reduced.

## Discussion

Previous serial OCT studies of STEMI patients have mainly focused on the chronic phases between 6 and 15 months post-procedure [7, 9, 15]. Recently, Amabile et al. investigated thrombus evolution during the very early phase (2–8 days after the onset) of acute coronary syndrome; however, the targeted lesions were treated without stenting [8]. To the best of the authors’ knowledge, this may be the first study to use FD-OCT to prospectively investigate the very early vascular responses to any type of stent implantation for STEMI lesions.

At 2 weeks after CoCr-EES implantation for the treatment of STEMI culprit lesions, the following vascular responses could be anticipated in the majority of cases (in comparison to the status immediately after the procedure): (1) reduced intra-stent thrombus; (2) lumen area enlargement; (3) modestly covered stent struts (almost 80%); (4) a decreased frequency of malapposed struts; and (5) reduced coronary dissection. Although fibrin deposition, residual thrombus and/or plaque prolapse, as opposed to neointimal/endothelial coverage, might be predominant at 2 weeks, modest strut coverage could be interpreted as a sign of vascular healing.

The present study revealed that various healing processes might occur more rapidly than was generally thought. There are several mechanisms that may contribute to this process. Basically, the thin stent strut design of CoCr-EES was proven to be associated with better healing responses in comparison to stents with thicker struts [16]. In addition, aggressive stent expansion under FD-OCT guidance might enhance early strut coverage and result in a low frequency of incomplete strut apposition, as was observed in this study. This meticulous approach is known to allow for extremely good strut “embedment” in the vascular wall, with a median depth of 84 µm per lesion in another study [17]; this is almost identical to the actual strut thickness of CoCr-EES. The sufficient embedment of struts, which are mostly embedded in the vascular wall, is expected to be correlated with the extent of future strut coverage. Despite the modest tissue coverage of the stent struts—which reduce the diameter of the lumen—the lumen dimensions were substantially increased at 2-weeks post-implantation, which indicates that intra-stent thrombus was further reduced during this period. With regard to the improvement of edge dissection, proximal dissections appeared to resolve earlier. In fact, this study demonstrated that the dissections of 3 of 3 cases disappeared within just 2 weeks. The blood flow direction, which continuously lifts the proximal dissection flap up toward the vascular wall and which also impairs drug elution into the proximal adjacent segment, may act favorably to achieve early healing of stent edge dissection.

### Antithrombotic efficacy of drug-eluting stents by drug and polymer type

Despite the thrombus-rich environment, this study demonstrated a significant reduction of in-stent thrombus within 2 weeks; this corresponded to a substantial period in which the risk of thrombotic complications was increased after coronary stenting. The present study indicates that the thrombus size can be expected to decrease by an average of approximately 50% (with 60% of the length and 80% of the area of the thrombus preserved inside stent) at 2 weeks after CoCr-EES implantation.

The high antithrombotic potential of the fluorinated polymer of CoCr-EES has been indicated by several experiments [3, 18], which partially accounts for the striking reduction of in-stent thrombus in this study, as well as the low rate of stent thrombosis in other clinical studies [4, 5, 19]. Furthermore, in terms of drugs, the other important component of a drug-eluting stent, a biolimus-eluting stent with a bare metal (non-polymer) luminal surface was found to reduce thrombotic events in comparison to bare metal stents [20], indicating that exposure of the vascular wall to biolimus has an independent inhibiting effect on thrombus formation. Thus, both the polymer and drug components of drug-eluting stents have independent antithrombotic effects, and optimal combinations may be advantageous for preventing early stent thrombosis.

### Clinical implications

The present results support the current expert consensus on the treatment of STEMI. This study described the approximate temporal changes after stent implantation during emergent procedures, which should be extremely helpful for physicians. Furthermore, the potential of FD-OCT-guided techniques were revealed. Because high-resolution FD-OCT enables the precise visualization of stent expansion, strut apposition, strut embedment, and the residual thrombus burden during the procedure [21, 22], it may be associated with accelerated vascular healing reactions within the initial 2 weeks. However, to confirm this hypothesis, it will be necessary to perform further randomized clinical studies to compare the vascular responses after stent implantation with or without FD-OCT guidance.

Furthermore, the findings may contribute to the ongoing debate about the optimal strategies of dual antiplatelet therapy, which have been the greatest concerns in the clinical arena. The vast majority of patients in this study received clopidogrel, which was mostly identical to the EXAMINATION study [4]; this prompts speculation as to what happened in that monumental study. Currently, other regimens that include prasugrel or ticagrelor are more frequently used in the actual clinical setting. This factor is likely to have a different impact on the clinical outcome [23, 24]. Since the publication of key study findings [25], prolonged dual antiplatelet therapy remains the recommended strategy. Nevertheless, the drug regimen should be repeatedly re-evaluated and altered based on the elevated bleeding risk. Currently, several prospective, multicenter, randomized controlled clinical trials, such as the GLOBAL LEADERS trial [26] and the STOPDAPT 2 study, in which dual antiplatelet therapy is continued for 1 month, are ongoing. The results of the present study, which focus on the safety mechanisms of drug-eluting stents, should be incorporated into comprehensive clinical trials for future constructive discussions on shortening the duration of dual antiplatelet therapy.

### Study limitations

The population of the present study was relatively small. However, considering the extensive effort required for FD-OCT analysis, the sample size was at the most attainable goal. In this respect, it was similar to the goals of previous investigations. Another limitation is the lack of a control group for statistical comparisons. Although direct comparison with patients who received bare metal stents would be ideal, designing a head-to-head comparison between bare metal stent (BMS) and drug-eluting stents is ethically difficult due to strong clinical evidence [4, 5, 20]. Furthermore, similar vascular responses might be observed if other DESs or BMSs were applied in this study, as indicated in our previous pilot study, which included a very small number of patients (*n* = 6 for each) [11]. However, considering the significant reduction in the incidence of clinical thrombotic events within the initial 2 weeks in the EXAMINATION study, the early vascular responses of patients who receive a CoCr-EES might be found to differ from those of patients who receive a BMS if an adequate number of patients could be enrolled. A third restriction is a potential selection bias due to the small number of patients who were eligible for FD-OCT evaluation during emergent procedures: the enrolment of cases with severe hemodynamic conditions tended to be avoided. Moreover, the lesion morphology and site must be suitable for FD-OCT evaluation. This study mainly included patients with multi-vessel disease because FD-OCT assessments were scheduled during staged PCI. A fourth limitation is that it might be difficult to accurately distinguish thrombus from tissue prolapse because white (platelet) thrombus can also be detected as a homogenous high signal without attenuation. In the present study, thrombus was defined as a mass of ≥ 250 μm in height with an irregular surface, protrusion into the lumen, and significant attenuation behind the mass. Accordingly, some white thrombi might have been misclassified as protrusions in the present study. However, the classification methods for thrombi and protrusions that we applied had previously been established by Soeda et al. [21], whose findings are considered to be important for predicting long-term clinical outcomes. Furthermore, the combination of such intra-stent tissues was significantly reduced from after intervention to the 2-week follow-up examination, which is consistent with the temporal changes in thrombi that were defined in this study. Finally, although several definitions of OCT parameters have been reported [10, 21], we decided to perform our analysis using a pre-specified definition based on the pilot study. The results of clinical studies are affected to varying degrees by differences in definitions or the sampling rates of cross-sections [27]; however, the serial data set that was used in the present study was considered to substantially minimize the influences on vascular behaviors.

## Conclusions

This study revealed the vascular responses in the early phase after the treatment of STEMI lesions with a second generation drug-eluting stent. The responses included reduced thrombus, lumen enlargement, the earlier progression of strut coverage, and the improvement of strut apposition and dissection. The combination of these factors may be responsible for the safety of CoCr-EES within the initial 2 weeks.
